# Valvular Disorders in Carcinoid Heart Disease

**DOI:** 10.5935/1678-9741.20160079

**Published:** 2016

**Authors:** Shi-Min Yuan

**Affiliations:** 1 MMed, PhD. The First Hospital of Putian, Teaching Hospital, Fujian Medical University, Fujian Province, China

**Keywords:** Carcinoid Heart Disease, Heart Failure, Pulmonary Valve Stenosis, Tricuspid Valve Insufficiency

## Abstract

Carcinoid heart disease is a rare but important cause of intrinsic right heart valve disorders leading to right heart failure. Occasionally, left-sided heart valves may also be involved. The characteristic cardiac pathological findings of carcinoid heart disease are endocardial thickening as a result of fibrous deposits on the endocardium. Echocardiographic examination and right heart catheterization are very useful for the diagnosis of the lesion. If more cardiac valves are affected, multiple valve replacement should be considered. The management of the pulmonary valve lesion depends on the extent of the diseased valve, either by valvulotomy, valvectomy, or valve replacement. Percutaneous valve implantations in the pulmonary and in the inferior vena cava positions have been advocated for high-risk patients.

**Table:** 

Abbreviations, acronyms & symbols	
5-HT	**= 5-hydroxytryptamine**
5-HIAA	**= 5-hydroxyindoleacetic acid**

## INTRODUCTION

Carcinoid heart disease, also known as Hedinger syndrome, is rare affecting at least 20% of patients with carcinoid syndrome. It is considered as a result of high circulating hormone levels, mainly serotonin, impacting on the heart, leading to most commonly valvular fibrosis^[1]^. It is an important cause of intrinsic right heart valve disorders with significant morbidity and mortality secondary to right heart failure^[2]^. Cardiac involvement in carcinoid disease generally results in right-sided valvular lesions, accompanied by tricuspid insufficiency and pulmonary stenosis. The typical valvular lesions of carcinoid heart disease include both tricuspid regurgitation and pulmonary valve stenosis, and the evolving right heart failure may lead to more prominent symptoms^[3]^. In addition, Materazzo et al.^[4]^ and Le Métayer et al.^[5]^ reported left-sided cardiac involvement in carcinoid disease.

## PATHOLOGY

Carcinoid heart disease are caused by endocardial deposition of pearly fibrotic plaques (notable for absence of elastic fibers) that generally extend to the right-sided valves, leading to multiple patterns of severe valve dysfunction. Plaque formation causes annular constriction, leaflet thickening, and fusion of the subvalvular apparatus. Marked degeneration of the leaflet architecture leads to severe retraction and noncoaptation of the valve^[6]^. Typically, carcinoid heart disease involves the right-sided endocardium, valves and subvalvular apparatus^[1]^. The deposition of carcinoid plaques, fibrous tissue, myofibroblasts and collagen results in annular constriction, leaflet thickening and retraction, and subvalvular fusion and shortening^[7]^. Tricuspid regurgitation was almost universal at 92-100%, followed by tricuspid stenosis (38-44%), pulmonary regurgitation (31-38%), and pulmonary stenosis (25-31%). The prevalence of left-sided disease was 0-39%, and most of the advanced right-sided cardiac abnormalities had a lower frequency of left-sided disease than did those with mild right-sided abnormalities^[8]^. Luis et al.^[9]^ defined the proportions of the valve pathologies of carcinoid heart disease (Table 1).

**Table 1:** Relative proportions of valvular pathologies in carcinoid heart disease.

Heart valve	Most common	Less common
Tricuspid valve	Isolated regurgitation	Mixed stenosis & regurgitation
Pulmonary valve	Mixed regurgitation & stenosis	Isolated regurgitation, or, isolated stenosis
Left sided valve	Isolated regurgitation	

The characteristic pathological findings are endocardial plaques of fibrous tissue that may involve the tricuspid valve, pulmonary valve, cardiac chambers, venae cavae, pulmonary artery, and coronary sinus. The fibrous reaction may involve not only the valve leaflets, but also the subvalvar apparatus, including the tendinous chords and papillary muscles of the tricuspid valve, and more rarely the mitral valve in cases with left sided involvement. The fibrous tissue in the plaques results in distortion of the valves leading to either stenosis, regurgitation, or both. The preferential right heart involvement is most likely related to inactivation of the vasoactive substances by the lungs. In the 5-10% of cases with left-sided valvar pathology, one should suspect either extensive liver metastases, bronchial carcinoid, or a patent foramen ovale^[2]^. Cardiac carcinoid plaques have been related to the exposure of the right heart to serotonin and other tumor byproducts released from hepatic metastases^[10]^. Ninety-seven percent of the patients with cardiac involvement had tricuspid valve disease, of whom 90% displayed moderate or severe tricuspid regurgitation; smaller numbers had coexistent tricuspid stenosis. Pulmonary valve pathology was noted in 88% of patients, of whom 81% had pulmonary regurgitation, 53% had pulmonary stenosis, and only 7% had left-sided involvement, in whom the most typical feature was mild-to-moderate mitral regurgitation^[2]^.

In carcinoid heart disease, the characteristic cardiac pathological findings are endocardial plaques of fibrous tissues^[11]^. Usually, only carcinoid tumours that invade the liver result in pathological changes to the heart. The cardiac manifestations are caused by the paraneoplastic effects of vasoactive substances, such as 5-hydroxytryptamine (5-HT or serotonin), histamine, tachykinins, and prostaglandins released by the malignant cells rather than any direct metastatic involvement of the heart. Ordinarily, the vasoactive tumor products are inactivated by the liver, lungs, and brain, but the presence of hepatic metastases may allow large quantities of these substances to reach the right side of the heart without being inactivated by the liver^[2]^. Serotonin is a common product of carcinoid tumors and it contributes to cardiac failure as a result of fibrous deposits on the endocardium. Most such tumors contain tryptophan hydroxylase, which is able to produce serotonin after hydroxylation and subsequent decarboxylation of the tryptophan. 5-Hydroxyindoleacetic acid (5-HIAA) is the main metabolite, an oxidation product, of serotonin. In urine samples, 5-HIAA can be detected for the determination of serotonin levels in the body. There is a significant correlation between carcinoid heart disease and elevated urinary 5-HIAA excretion, which is indicative of serotonin production. However, non-carcinoid heart disease patients reported in literature have significantly lower serotonin release^[12]^. Zuetenhorst et al.^[13]^ also found that median levels of N-terminal pro B-type natriuretic peptide and 5-HIAA were significantly higher in patients with carcinoid heart disease compared to those without (894 ng/L *vs.* 89 ng/L, *P*<0.001; 815 mmol/24 hours *vs.* 206 mmol/24 hours, *P*=0.007), but no significant differences were detected in atrial natriuretic peptide levels. Dilation of the right atrium and ventricle as well as thickening of the tricuspid valve and degree of regurgitation were statistically significant correlated with N-terminal pro B-type natriuretic peptide levels. Patients in the progression of carcinoid heart disease with a cardiac score increase >25% had a significant higher urinary peak 5-HIAA levels than those <25% (265 mg/24 hours *vs.* 189 mg/24 hours; *P*=0.004)^[10]^. Patients with carcinoid syndrome therefore have raised concentrations of 24 hour urinary 5-HIAA. In a large series of patients with carcinoid syndrome and cardiac involvement the mean 24 hour urinary excretion of 5-HIAA was 10-fold higher than the reference value (reference value, 50 mmol/L/24 hours). Some studies suggest that a positive correlation exists between urinary concentrations of 5-HIAA, disease progression and worsening prognosis. This has been attributed to the fact that higher circulating concentrations of vasoactive substances produced by the tumor (especially 5-HT causing fibroblast proliferation) are likely to induce more cardiac damage. In patients with both right- and left-sided carcinoid heart disease, urinary values of 5-HIAA appear to be higher in patients without interatrial shunts compared to those with interatrial shunts^[2]^.

## DIAGNOSIS

The characterized triad symptoms of carcinoid syndrome are cutaneous vasomotor flushing, gastrointestinal hypermotility and cardiac involvement^[14]^. Symptoms of flushing and diarrhea were almost threefold more common in the cardiac group. Circulating serotonin levels were more than twofold higher in the cardiac group. Urine levels of the serotonin metabolite 5-HIAA were almost fourfold higher in the heart disease patients compared with the noncardiac group (219±124 mg/24 hours *vs.* 55.3±141 mg/24 hours, *P*<0.001). Elevated serum serotonin (above the upper limit of normal range, *i.e.*, >1500 µmol/mL) was 100% sensitive but only 46% specific for carcinoid heart disease^[8]^. Carcinoid tumors secrete a variety of agents, including kallikrein, histamine, prostaglandins, adrenocorticotropic hormone, gastrin, calcitonin, and growth hormone; however, serotonin has received the most attention in hypotheses concerning the genesis of heart disease. Elevated urinary 5-HIAA is a less-than-ideal serotonin surrogate because it misses 10% of patients with true elevated circulating serotonin levels^[8]^. These symptoms are caused by the release of vasoactive substances, mainly 5-HT as well as 5-hydroxytryptophan, histamine, bradykinin, tachykinins, and prostaglandins^[15]^. Tricuspid valve disease (especially regurgitation) was most prevalent. The left-sided valve lesions tended to be less common and of milder severity than their right-sided counterparts. Cardiac catheterization and echocardiography had an overall concordance of 91% on the basis of valvular lesions^[8]^. Some 92% had New York Heart Association class III or IV symptoms of congestive heart failure, and 8% had class II symptoms. Right-sided filling pressures were mildly to moderately elevated, whereas left-sided filling pressures were generally normal^[8]^. In such patients, physical examination usually reveals a systolic murmur along the left sternal edge, produced by tricuspid regurgitation; concomitant murmurs of pulmonary stenosis or regurgitation may also be present^[2]^. In many patients, the structural valvular lesions will lead to symptomatic right-sided heart failure (edema, hepatomegaly, fatigue with exertion, and low cardiac output)^[10]^. The diagnosis of carcinoid syndrome is usually suspected by the clinical features and confirmed by identification of the primary tumor, localization of metastatic lesions, and detection of increased urinary excretion of the by-product of serotonin metabolism, 5-HIAA^[16]^. Laboratory tests may reveal an elevated *γ*-glutamyl transpeptidase due to liver metastases^[17]^. Patients with carcinoid heart disease typically present with symptoms of right heart failure (hepatomegaly, edema, ascites, fatigue and low cardiac output)^[6]^. The diagnosis of carcinoid heart disease can be delayed as hepatic dysfunction and may conceal the signs and symptoms of right heart failure^[17]^. Right ventricular enlargement and decreased right ventricular function can also be noted on echocardiography^[18]^. Echocardiographic examination and right heart catheterization are very useful in defining the disease. Catheterization confirms severe tricuspid incompetence with a v-wave in the right atrium and marked regurgitation evident on right ventricular angiography^[3]^. Characteristic echocardiographic findings include endocardial thickening and lack of compliance of the right ventricle, in addition to the typical thickening of both pulmonary and tricuspid valves, with significant pulmonary stenosis and tricuspid insufficiency^[17]^. The echocardiographic visualization of carcinoid heart disease is listed in Table 2.

**Table 2:** Echocardiographic findings of carcinoid heart disease^[1,8,9]^.

Echocardiographic technique	Echocardiographic findings
	Leaflet: thickening, retraction, reduced mobility, laminar regurgitant flow
	Valve: incomplete coaptation
Two-dimensional	Right ventricular free wall: thickening Small pericardial effusions
	Right atrium and (or) right ventricle enlargement (81%)
	Thickened immobile tricuspid valve (56%)
	"Dagger" shaped Doppler profile with early peak & rapid decline (severe tricuspid regurgitation)
	Typical parabolic profile (less severe tricuspid regurgitation)
Continuous Doppler	Increased regurgitant flow (2.6±0.5 m/s; range, 1.5-4.0 m/s)
	Prolonged pressure half-time (116 ± 43 ms; range, 45-200 ms) (tricuspid regurgitation)
	Steep pressure half-time (pulmonary regurgitation)
Color Doppler	Colored reverse flow

## TREATMENT

Commonly diuretic therapy and supportive therapy are essential for peripheral edema, hepatic congestion and ascites^[9]^. Medical treatment include somatostatin analogs (such as, octreotide and lanreotide) for reducing urinary 5-HIAA, cytotoxic chemotherapy for extensive disease and dual endothelin receptor antagonist (such as, bosentan) for preventing valvular and mural fibrosis and improving heart function^[9]^. The somatostatin analogues act by binding to somatostatin receptors, inhibiting secretion of tumor byproducts, and alleviating symptoms in the majority of patients^[10]^. Relief of symptoms can be achieved surgically by debulking the tumor, and sometimes, in those with hepatic metastases, by hepatic artery ligation or embolization^[2]^.

The surgical indications of carcinoid heart disease are, 1) symptomatic right ventricular failure; 2) severe valvular dysfunction; 3) systemic venous pressure elevation^[6]^; and 4) as a prelude to planned surgical hepatic tumor resection^[9]^. Whereas, the contraindications are, 1) end-stage metastatic disease, and 2) patients with poorly controlled carcinoid symptoms despite octreotide therapy, or hepatic dearterialization^[15]^. When the pulmonary insufficiency is mild and very well tolerated and when the left heart filling pressures are normal, pulmonary valve replacement is unnecessary. The optimal surgical approach to right-sided valvular lesions remains a subject of debate. Tricuspid valve replacement has been accepted by most centers, however, the management of pulmonary valvular disorder remains debatable. If pulmonary valve regurgitation is left untreated, there would be right ventricular overload in the long run; whereas in patients with pulmonary valve replacement, the postoperative recovery appear to be more favorable^[6]^. By comparison, the limitations for homograft use for pulmonary valve replacement are homograft constriction, homograft calcification, homograft plaque deposition; while the advantages of bioprosthesis are remarkable in better short-term outcomes, longer valve durability, free of anticoagulate uptake and uncommon carcinoid involvement^[15]^. When the pulmonary and tricuspid valves are both involved, open pulmonary valvulotomy is a reasonable option. If more cardiac valves are affected, multiple valve replacement should be considered. In most cases, involvement of the valve leaflets is too extensive to allow surgery for commissurotomy or valvotomy, and pulmonary valvectomy surgery is required. Narine et al.^[19]^ recommend the use of mechanical prostheses in carcinoid heart disease with a concern of the fibrous degeneration of the bioprosthetic valves. Some reports of surgical treatment of tricuspid insufficiency due to carcinoid disease describe use of mechanical prostheses^[20,21]^, and pulmonary valve replacement has been advocated^[22]^. Due to the increased risk for repeat surgical valve replacement, percutaneous stent implantation into the pulmonary artery, followed by the implantation of a balloon expandable transcatheter heart valve is warranted. A simplified approach including pulmonary valvectomy and bioprosthetic tricuspid valve replacement surgery are the procedures of choice. While surgical valve replacement is currently the gold standard treatment for symptomatic carcinoid valve disease, transcatheter pulmonary valve replacement should be considered as an alternative approach in high-risk candidates^[18]^. Percutaneous valve implantation in the pulmonary and in the inferior vena cava positions may offer a novel, minimally invasive option avoiding open-heart surgery in the high-risk patients^[23]^. The Medtronic Melody^®^ valve and the Edwards SAPIEN^®^ valve are in use today for percutaneous pulmonary valve implantation (PPVI) in children and in adults with congenital heart disease^[24,25]^. Surgical complications can include bleeding, carcinoid crises, right ventricular dysfunction, cardiac conduction disorders, renal failure and sepsis^[9]^.

## DEBATES

Contrary to conventional guidelines of heart valve operation and choices of heart valve prosthesis^[26,27]^, most surgeons prefer bioprostheses in the tricuspid position because of the lower propensity to thrombosis in carcinoid heart disease. However, questions have been raised about the long-term durability of porcine valves exposed to the metabolic products of carcinoid tumors. Progression onto a pulmonary valve homograft and onto a tricuspid bioprosthetic valve has been reported^[28,29]^. While most surgeons would prefer to use a bioprosthetic valve for replacement of the tricuspid valve, many reports of the surgical treatment of carcinoid heart disease describe the use of mechanical valves, because of the concern about secondary development of carcinoid lesions in bioprostheses. An antibiotic sterilized aortic homograft can be a candidate prosthesis for pulmonary valve replacement^[3]^. In addition, pulmonary valvotomy or valvectomy, as opposed to pulmonary valve replacement, remains controversial^[3]^. Patients who have no contraindication for valve surgery and whose cardiac symptoms develop at an early stage of the disease should undergo operation. The comparisons of the valve prosthesis of choice are shown in Table 3.

**Table 3:** Advantages and disadvantages of valvular prostheses of choices for carcinoid heart disease patients^[6,15,28]^.

Valvular Prosthesis	Advantage	Disadvantage
Mechanical	No carcinoid involvement.	Long-term coagulation therapy
	Better short-term outcomes	
	Valve durability	Carcinoid plaque deposition
Bioprosthesis	Uncommon carcinoid involvement	Prosthetic degeneration
	Avoidance of long-term warfarin use & secondary coagulopathies	Organizing thrombus
		Short durability
Bioprosthesis (stentless)	No need of long-term coagulation therapy	Potential of restenosis
		Higher incidence of reintervention
		Homograft constriction
Homograft	No need of long-term coagulation therapy	Homograft calcification Plaque deposition
		Premature dysfunction with accelerated stenosis

## OUTCOMES

Severity of tricuspid valve regurgitation was an important predictor of outcome^[10]^.

In these patients with advanced metastatic carcinoid disease, prognosis was poor, with a median survival of only 2.6 years after diagnosis of cardiac involvement. The severity and hemodynamic consequence of carcinoid heart disease contributes to the high mortality^[10]^. Analysis of 17 series with carcinoid heart disease patients who had undergone surgical biological tricuspid valve replacement revealed a 30-day mortality of 20%. It was suggested that biological valves have an acceptable lifespan in carcinoid heart disease^[30,31]^. Long-standing severe pulmonary valve regurgitation, as seen after pulmonary valvectomy, may have a detrimental effect on right ventricular remodeling^[15]^. Schoen et al.^[32]^ reported a case of carcinoid heart disease, in which they observed carcinoid plaque that involved the tricuspid bioprosthesis at postmortem examination. The 3-year mortality for patients with carcinoid heart disease showed a 31% survival rate, significantly lower than that of patients without cardiac involvement^[2]^. Conradi et al.^[33]^ reported transcatheter pulmonary valve replacement resulted in a favorable acute outcome in both of their patients, with adequate valve function and good heart function. The patients tolerated the procedure well without any complications. The perioperative mortality rate was reduced from >20% to currently <10%^[10]^. The overall operative mortality was 10%^[15]^. The survival between the cardiac and noncardiac groups was not statistically different (survival from onset of symptoms: 13.6 years *vs.* 9.7 years; survival from time of diagnosis: 4.8 years *vs.* 7 years)^[8]^.

## CONCLUSION

Carcinoid heart disease is a rare cause of right-side heart valve disease. Echocardiography remains a reliable method for the definite diagnosis. Valve replacement is currently the gold standard treatment for symptomatic carcinoid valve disease. However, debates remain in terms of the choice of heart valve prosthesis. The surgical management of the pulmonary valve relies on the pathological extent and patient's condition. Transcatheter pulmonary valve replacement should be considered as an alternative approach in high-risk candidates.

**Table:** 

Authors’ roles & responsibilities	
SMY	Study conception and design; analysis and/or interpretation of data; manuscript writing; final approval of the manuscript
